# Total Intravenous Anesthesia and Acute Normovolemic Hemodilution for Sarcoma Surgery

**DOI:** 10.7759/cureus.11319

**Published:** 2020-11-03

**Authors:** Vincent Y Ng, Kimberly N Hollander, Shamus R Carr, Kenichi Tanaka

**Affiliations:** 1 Department of Orthopaedics, University of Maryland School of Medicine, Baltimore, USA; 2 Greenebaum Comprehensive Cancer Center, University of Maryland Medical Center, Baltimore, USA; 3 Department of Anesthesiology, University of Maryland School of Medicine, Baltimore, USA; 4 Division of Thoracic Surgery, Department of Surgery, University of Maryland School of Medicine, Baltimore, USA

**Keywords:** intravenous anesthesia, normovolemic hemodilution, sarcoma

## Abstract

Despite optimal local control for high-risk soft tissue sarcomas (STS) with radiation and surgery, there are no other interventions that clearly and significantly reduce the risk of distant relapse after resection. Cytotoxic chemotherapy for localized STS is controversial and is associated with significant side effects. There are significant biologic perturbations that occur at the time of operation and numerous studies have demonstrated that surgical removal of the primary tumor can accelerate the growth of subclinical metastases. While the exact etiology of this phenomenon is unknown, there is some evidence to suggest that allogeneic blood transfusion and volatile inhaled anesthetics may be associated with tumor-promoting processes. At our institution, we have utilized acute normovolemic hemodilution and total intravenous propofol-based anesthesia to avoid these potentially detrimental factors.

## Introduction

There are approximately 13,000 Americans diagnosed with soft tissue sarcoma (STS) and 5,000 who succumb to metastatic sarcoma per year [1]. Currently, there are no available therapeutics to reliably effect a cure if patients develop distant disease, usually in the form of pulmonary metastases. Although most patients present with large primary tumors and have a long duration of pre-diagnosis symptoms, radiologically evident pulmonary metastases are not detected during the initial work-up. Nevertheless, for large, localized, high-grade, trunk, and extremity STS, only about 50% of patients can be definitively cured [1]. The standard treatment for high-risk STS is radiation plus R0 surgical resection. Other neoadjuvant or adjuvant treatments such as cytotoxic chemotherapy, molecular targeted therapeutics, and checkpoint inhibitor immunotherapy either are experimental or controversial in reducing the risk of distant relapse after resection of the localized primary tumor.

After resection of the primary STS, the risk of distant relapse is highest within the first one to two years and outweighs the risk of local recurrence by several-fold. Although surgical removal of the primary tumor is a requisite for achieving a cure, it has the unfortunate effect in some patients of accelerating growth of pre-existing subclinical pulmonary nodules or establishing a favorable premetastatic niche for circulating tumor cells to colonize. Various studies in animals have found that this phenomenon is associated with soluble factors released by the primary tumor, immune-mediated suppression of secondary tumor sites, and growth-favorable systemic conditions promoted by the act of surgery itself [2,3]. In addition, there are events in the perioperative period that cause significant biologic perturbations and may play a role in increasing the risk of pulmonary metastases. Recognition of these events and making minor adjustments at the time of surgery may attenuate their effect.

At our institution, we have begun to utilize the Propofol-based total intravenous anesthesia (TIVA)/ acute normovolemic hemodilution ANH protocol during surgery for patients with high-risk trunk and extremity sarcomas. This is the first published description of this technique in this population to our knowledge. In our experience, it has proven to be safe and effective. The long-term oncologic outcomes will be the subject of future study. We have avoided the use of epoietin or a cell-saver device in sarcoma patients due to the conceivable risks of promoting tumor growth and disseminating tumor cells, respectively. The following is an example of TIVA/ANH in our practice.

## Case presentation

**Figure 1:**
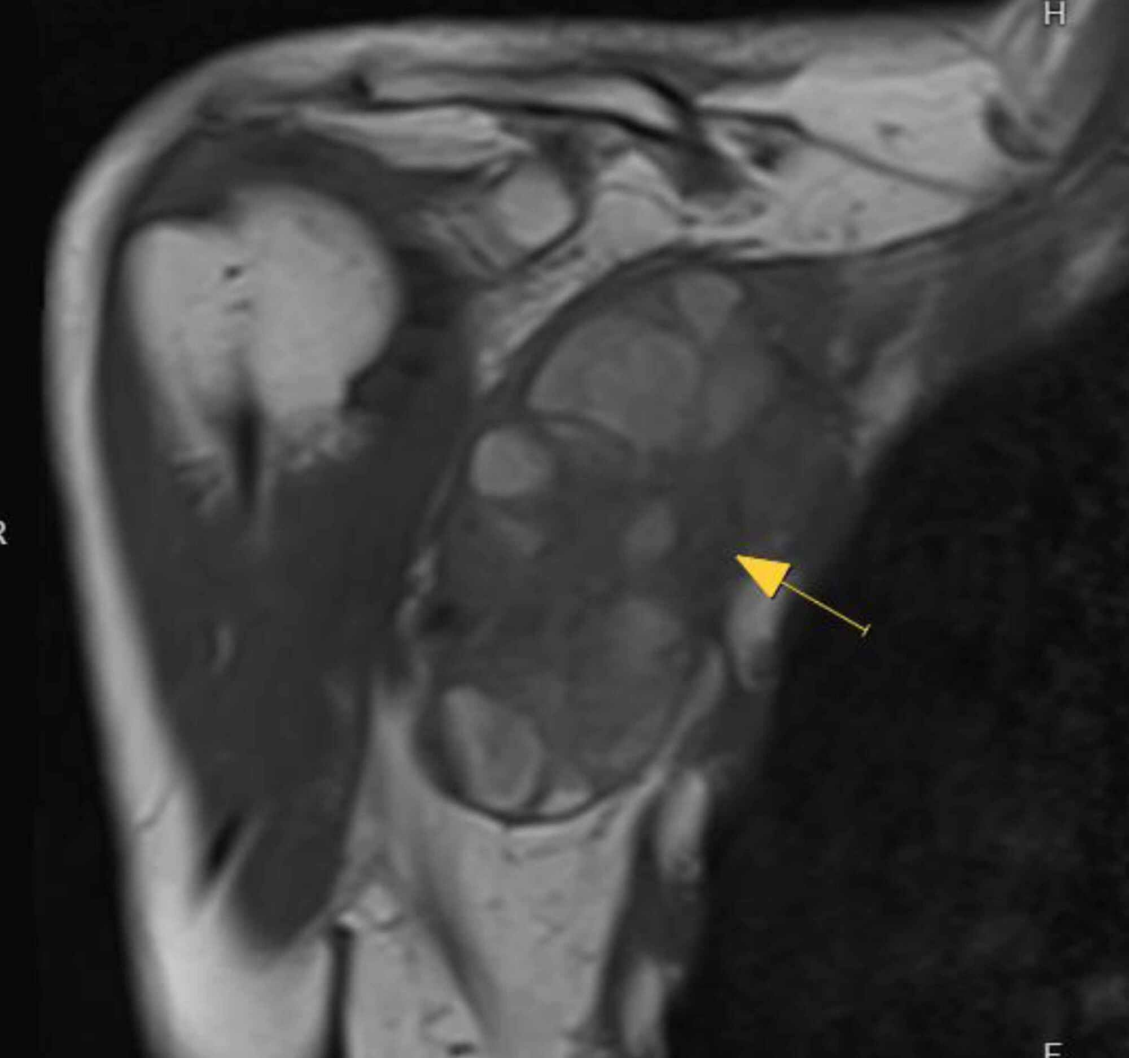
Coronal T1 magnetic resonance imaging of a right axillary malignant peripheral nerve sheath tumor (MPNST).

A 47-year-old male, otherwise healthy, was diagnosed with a localized 9.8 cm high-grade malignant peripheral nerve sheath tumor (MPNST) in his right axilla involving his brachial plexus and subclavian artery (Figure 1). He was 5’10” and weighed 68 kg. He received neoadjuvant radiation and concomitant chemotherapy. His preoperative Hemoglobin (Hgb) was 13.1 g/dL (ref: 12.6 - 17.4 g/dL). A central line and peripheral IVs were established and 600 ml of autologous blood was drawn, lowering his Hgb to 12.0 g/dL. With hemodilution and an estimated blood loss of 450 ml, his Hgb fell to 9.5 g/dL at mid-surgery and 8.9 g/dL at the conclusion of surgery. He received a total of 4.5L of crystalloid and the 600 ml of autologous blood was reinfused during wound closure. The duration of surgery was approximately six hours and included dissection of the tumor en-bloc away from numerous major nerves and an end-to-end vascular graft anastomosis for the axillary artery. The final margins were R0. The Hgb on postoperative day (POD) one was 10.5 g/dL and he was discharged on POD two with a Hgb of 11.0 g/dL. The patient consented to the publication of the details of his case.

## Discussion

Propofol-based total intravenous anesthesia (TIVA) 

Volatile inhaled anesthetic agents such as sevoflurane, desflurane, and halothane are increasingly being used in the hospital and ambulatory surgery settings. They are introduced via the arterial blood through the pulmonary circulation and allow for rapid induction of sedation and rapid postoperative recovery. Propofol-based total intravenous anesthesia (TIVA) is less commonly used than inhalational anesthesia, but considerations for it include reducing postoperative nausea, vomiting, and delirium, minimizing the risk of malignant hyperthermia, preventing intracranial hypertension, and performing surgery in military settings. Propofol is more expensive, is much less commonly used, and requires closer monitoring during surgery than inhaled anesthetics.

From an oncologic standpoint, there is some rationale to favor TIVA over inhaled volatile anesthetics. Compared to TIVA, volatile anesthetics have been shown to have deleterious effects on natural killer cell function and increased pulmonary metastases [4]. Hypoxia-inducible factor-1α (HIF-1α) is upregulated with isoflurane, leading to increased proliferation and migration of malignant solid tumor cells, while propofol inhibited HIF-1α and its downstream effects [5,6]. Furthermore, insulin growth factor-1 (IGF-1) and vascular endothelial growth factor (VEGF) are increased and associated with cell migration and the production of matrix metalloproteinases [7]. Volatile anesthetics have a largely immunosuppressive effect also mediated by macrophages, T-cells, and dendritic cells. Propofol, in contrast, has shown beneficial effects from an oncologic standpoint through anti-inflammatory and antioxidant properties and maintenance of natural killer cell function [8,9]. Proliferation, invasion, and pulmonary metastasis of osteosarcoma cells in small animal models are inhibited by propofol [10,11]. These effects have been shown across a variety of other solid tumor types including melanoma and lung carcinoma. A large retrospective analysis of over 7,000 cancer patients who underwent surgery found that volatile inhalational anesthesia was associated with a hazard ratio of almost 1.5 after multivariate analysis compared to TIVA [12]. Although there is some evidence demonstrating no significant difference between TIVA and volatile anesthesia, this discrepant data may be reconciled by assessing it in the clinical context and concluding that the oncologic benefit of TIVA is most pronounced in patients with larger tumor resections compared to minimally invasive or minor surgeries [13]. Further studies are warranted to evaluate if STS exhibit similar biologic behavior as these other malignancies in response to propofol.

Acute normovolemic hemodilution (ANH)

Due to the magnitude of surgery to resect STS, intraoperative blood loss can sometimes lead to the need for blood transfusion. Perioperative blood transfusion has been reported to be associated with increased rates of recurrence and decreased survival for patients with osteosarcoma and STS [14,15]. An experimental sarcoma model in mice demonstrated significantly higher rates of tumor growth after perioperative blood transfusion with allogeneic blood compared to syngeneic (genetically and immunologically compatible) or autologous blood [16]. The independent effect of perioperative allogeneic blood transfusion in patients with STS, however, is controversial. 

There is scientific reason to support a strategy to minimize stored allogeneic blood transfusion in cancer patients. Transfusion-related immune modulation (TRIM) is mediated by a reduction in natural killer cell activity and interleukin-2 levels, an increase in pro-tumoral growth factors, and an infusion of incompatible major histocompatibility complex antigens. Leukoreduction of packed red blood cells (RBC) is routine in order to minimize post-transfusion infections, prevent febrile transfusion reactions, and decrease the likelihood of human leukocyte antigen (HLA) antibody formation, but the role that leukocytes play is not clear in TRIM. A rat model demonstrated that blood transfusion led to a fourfold increase in lung tumor retention and doubling in tumor mortality. Interestingly, the critical determinant of this effect was the length of blood storage prior to transfusion, and furthermore, the presence of aged erythrocytes rather than leukocytes or soluble factors mediated the effect [17]. 

While restrictive blood transfusion strategies such as withholding blood unless Hgb <7 g/dL or Hgb 7-10 g/dL with tachycardia or dyspnea are generally medically tolerated by patients, it may not be ideal. Anemia has been shown to be associated with poor prognosis in patients with STS. Several studies have shown significantly higher disease relapse rates and cancer-related death in STS patients with low hemoglobin (Hgb) levels [18,19]. Using a strategy to minimize both postoperative anemia and allogeneic blood transfusion may be beneficial in STS patients. 

Acute normovolemic hemodilution (ANH) is a blood conservation technique that avoids the risks of allogeneic blood transfusion and the use of stored blood. It involves the removal of whole blood from an anesthetized patient via an arterial line, peripheral IV, or central line into the bag pre-filled with citrate phosphate dextrose. The amount of blood varies from one to three units based on the expected surgical blood loss, preoperative Hgb, and estimated total blood volume. Normovolemia is maintained by infusing crystalloid or albumin replacement fluid in a 1:1 ratio to maintain intraoperative hemodynamics while minimizing the risk of pulmonary or renal interstitial edema. The ANH protocol reduces the amount of Hgb and other blood components being shed into the surgical field, lowering the net blood loss, and the risk of RBC transfusion. If an intraoperative transfusion is urgently required or typically, during the closure of the wound, the autogenous blood is reinfused back into the patient. In major orthopedic surgery, ANH has been shown to be safe and to reduce the incidence of allogeneic blood transfusion [20]. 

## Conclusions

There are no proven adjuvant strategies to reduce the relatively high rate of post-resection distant relapse of the disease in patients with localized high-grade STS. However, certain perioperative events may impact long-term oncologic outcomes. Utilization of a TIVA/ANH protocol can reduce the potential risks associated with volatile inhalational anesthetics, postoperative anemia, and stored allogeneic blood transfusion. These interventions appear to be safe during resection of STS and may provide a small but beneficial difference in overall survival. Due to the clinical heterogeneity and relative rarity of STS, it will be difficult to prove a significant independent effect for TIVA/ANH. However, utilized in combination with other modalities in the neoadjuvant and adjuvant setting, TIVA/ANH at the time of resection may play a role in a larger comprehensive treatment approach for high-risk STS. 
